# Splice variants of the Ca_V_1.3 L-type calcium channel regulate dendritic spine morphology

**DOI:** 10.1038/srep34528

**Published:** 2016-10-06

**Authors:** Ruslan Stanika, Marta Campiglio, Alexandra Pinggera, Amy Lee, Jörg Striessnig, Bernhard E. Flucher, Gerald J. Obermair

**Affiliations:** 1 Division of Physiology, Medical University Innsbruck, 6020 Innsbruck, Austria; 2 Department of Pharmacology and Toxicology, University of Innsbruck, 6020 Innsbruck, Austria; 3 Department of Molecular Physiology and Biophysics, Otolaryngology Head-Neck Surgery, and Neurology, University of Iowa, Iowa City, IA 52242, USA

## Abstract

Dendritic spines are the postsynaptic compartments of glutamatergic synapses in the brain. Their number and shape are subject to change in synaptic plasticity and neurological disorders including autism spectrum disorders and Parkinson’s disease. The L-type calcium channel Ca_V_1.3 constitutes an important calcium entry pathway implicated in the regulation of spine morphology. Here we investigated the importance of full-length Ca_V_1.3_L_ and two C-terminally truncated splice variants (Ca_V_1.3_42A_ and Ca_V_1.3_43S_) and their modulation by densin-180 and shank1b for the morphology of dendritic spines of cultured hippocampal neurons. Live-cell immunofluorescence and super-resolution microscopy of epitope-tagged Ca_V_1.3_L_ revealed its localization at the base-, neck-, and head-region of dendritic spines. Expression of the short splice variants or deletion of the C-terminal PDZ-binding motif in Ca_V_1.3_L_ induced aberrant dendritic spine elongation. Similar morphological alterations were induced by co-expression of densin-180 or shank1b with Ca_V_1.3_L_ and correlated with increased Ca_V_1.3 currents and dendritic calcium signals in transfected neurons. Together, our findings suggest a key role of Ca_V_1.3 in regulating dendritic spine structure. Under physiological conditions it may contribute to the structural plasticity of glutamatergic synapses. Conversely, altered regulation of Ca_V_1.3 channels may provide an important mechanism in the development of postsynaptic aberrations associated with neurodegenerative disorders.

Dendritic spines, the primary postsynaptic compartments of glutamatergic synapses in neurons of the central nervous system (CNS), play a key role in the manifestation of neuronal plasticity and consequently in memory formation. It is therefore not surprising that disorders of the CNS, such as autism spectrum disorders (ASD), schizophrenia, intellectual disabilities, as well as neurodegenerative diseases including Alzheimer’s or Parkinson’s, go hand in hand with changes in the number and morphology of dendritic spines and thus altered synaptic structure^1^. In Parkinson’s disease (PD) and PD-like animal models, for example, the GABAergic striatal projection neurons undergo spine pruning (reviewed in ref. 2). Moreover morphological changes of dendritic spines and aberrant restoration of synaptic connections has been hypothesized to underlie the pathology of L-DOPA-induced dyskinesia, the major debilitating side effect in the treatment of PD^3^,^4^,^5^,^6^.

Morphology and function of dendritic spines are critically controlled by the local concentration of calcium^7^,^8^. Besides NMDA and calcium-permeable AMPA receptors, voltage-gated calcium channels provide the major regulated calcium-entry pathway in dendritic spines^9^. The L-type calcium channels (LTCCs) Ca_V_1.2 and Ca_V_1.3 are widely expressed in brain^10^ and are located in dendritic spines^11^,^12^,^13^,^14^. Among LTCCs Ca_V_1.3 channels are functionally unique as they activate at more negative membrane potentials^15^,^16^, making them particularly susceptible for controlling neuronal excitability and calcium-dependent regulation of neuronal development and disease (for reviews see^17^,^18^).

Alternative splicing of Ca_V_1.3 gives rise to a long (Ca_V_1.3_42_ or Ca_V_1.3_L_) and several short C-terminal splice variants (in particular Ca_V_1.3_42A_; Ca_V_1.3_43S_), which differ in their voltage-dependence of activation, open probability, and calcium-dependent inactivation^19^,^20^,^21^. Most importantly Ca_V_1.3 channels have been associated with altered dendritic spine morphology in animal models of dopamine depletion, which induce a PD-like phenotype (ref. 14; reviewed in ref. 22). Moreover, mutations in the gene encoding for Ca_V_1.3 calcium channels (CACNA1D) have been linked to ASDs^23^,^24^ and to a severe congenital multiorgan syndrome with primary aldosteronism, seizures, and neurologic abnormalities^25^,^26^.

The full length variant of Ca_V_1.3 contains a C-terminal class 1 PDZ domain-binding sequence which interacts with the PDZ domain of the postsynaptic scaffolding proteins shank^27^ and densin-180^13^. Interestingly, both proteins can augment currents through Ca_V_1.3 channels: densin-180, together with CaMKII, mediates calcium-dependent facilitation^13^ and shank confers G-protein mediated inhibition of L-type currents in striatal medium spiny neurons by D2 dopaminergic and M1 muscarinic receptors^28^. Like Ca_V_1.3, shank and densin have been implicated in the regulation of the morphology and stability of dendritic spines^29^,^30^,^31^,^32^ and in neurological disease^33^,^34^.

Taken together, several lines of evidence suggest important individual roles of Ca_V_1.3 channels, densin-180, and shank in the regulation of postsynaptic structure. Therefore we tested the hypothesis that functionally diverse Ca_V_1.3 splice variants and their modulation by densin-180 and shank1b differentially regulate dendritic spine morphology. Our experiments demonstrate that expression of the short Ca_V_1.3 splices or increased levels of densin-180 or shank1b co-expressed with full-length Ca_V_1.3 induce aberrant dendritic spine elongation, which correlates with increased Ca_V_1.3 currents in cultured hippocampal neurons. Thus, a shifted balance in Ca_V_1.3 channel regulation may be an important mechanism contributing to dendritic spine pruning and synapse loss observed in neuronal disease.

## Results

### Ca_V_1.3 calcium channel α_1_ subunits are located on dendritic spines

**Figure 1:**
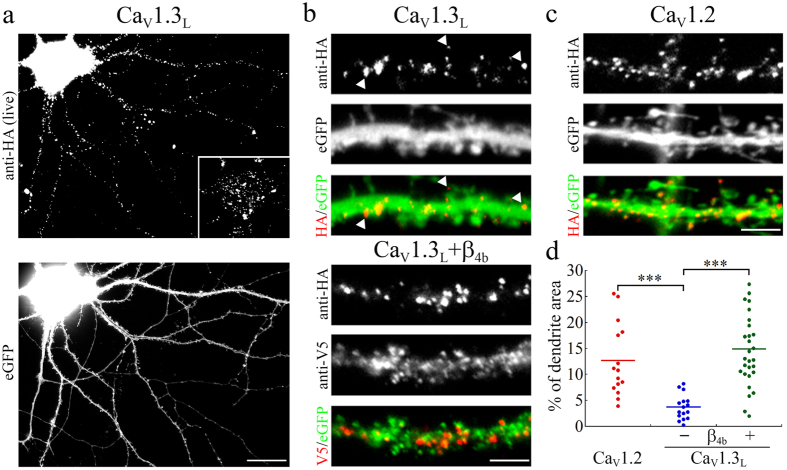
Membrane expression of the full-length extracellularly HA-tagged Ca_V_1.3_L_ calcium channel in hippocampal neurons. (**a**) Representative hippocampal neuron (21 DIV) transfected with the HA-tagged full-length Ca_V_1.3_L_ and labeled with anti-HA (live staining) and eGFP (to visualize cell morphology). Inset shows the cell soma at reduced contrast to visualize the clustered distribution of Ca_V_1.3_L_. (**b**,**c**) Labeling of Ca_V_1.3_L_ (**b**) on the dendritic surface showed similarly distributed clusters (examples indicated by arrowheads) as Ca_V_1.2 (**c**) on the shaft and on dendritic spines. Co-transfection of the auxiliary subunit β_4b_ (V5-tagged, anti-V5) increased total surface expression without changing the localization of Ca_V_1.3_L_ clusters ((**b**) lower panel). (**d**) Quantification of calcium channel surface expression. Total surface expression of Ca_V_1.3_L_ (total surface area on the dendritic shaft occupied by calcium channel clusters) is 29% of Ca_V_1.2. Co-expression of the β_4b_ subunit caused a 4-fold increase of Ca_V_1.3_L_ surface expression. Values for individual cells (dots) and the means (line) for each condition are shown. Statistics: ANOVA with Holm-Sidak posthoc analysis (F_2,57_ = 16.6; p < 0.001; posthoc: ***p < 0.001). Data from 2 culture preparations and 6 independent experiments, between 15 and 29 cells were analyzed in each condition. Scale bar, 20 μm (**a**) and 5 μm (**b,c**).

In order to investigate the precise subcellular localization of the human Ca_V_1.3 calcium channels we introduced an extracellular HA epitope into the loop connecting the IIS5-IIS6 domains. The HA-tag did neither influence the current properties nor the surface expression when expressed in tsA-201 cells (Supplementary Fig. S1). Similar to the distribution of rat Ca_V_1.3^13^,^13^ also human Ca_V_1.3_L_ displayed a clustered distribution on the somato-dendritic surface of cultured hippocampal neurons (Fig. 1a). The majority of Ca_V_1.3 surface clusters was located on the dendritic shaft but clusters were also found within dendritic spines (Fig. 1b, upper panel). When compared with the surface expression of Ca_V_1.2-HA channels (Fig. 1c), total Ca_V_1.3 surface expression was only 29% of that of Ca_V_1.2 (Fig. 1d). It is important to note that in our experiments in hippocampal neurons calcium channel α_1_ subunits are expressed without the ectopic expression of additional auxiliary subunits. Therefore plasma membrane localization of Ca_V_1.3-HA channels depends on the interaction with endogenous auxiliary subunits. Because β-subunit availability limits the surface expression of neuronal L-type channels, a hallmark of exogenously expressed L-type calcium channels is a marked increase in surface localization upon co-expression of heterologous β-subunits^36^. Indeed, co-expression of β_4b_ increased surface localization of Ca_V_1.3-HA approximately 4-fold (Fig. 1b, lower panel and Fig. 1d). This demonstrates that in hippocampal neurons Ca_V_1.3-HA expression is not saturated in the neuronal membrane. Rather Ca_V_1.3-HA competes with endogenous Ca_V_1.3 channels for β-subunits and thus the observed localization pattern should indeed reflect the distribution of endogenous Ca_V_1.3 channels.

**Figure 2:**
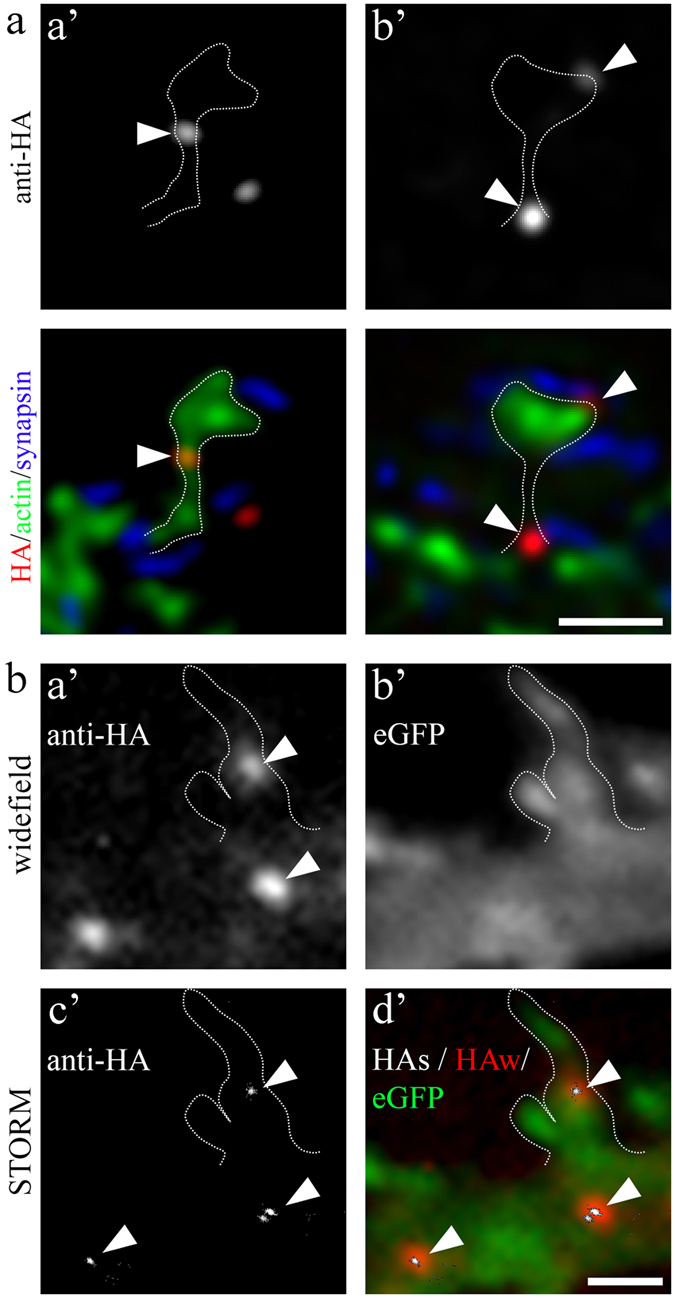
Superresolution microscopy of surface localized extracellularly HA-tagged Ca_V_1.3_L_ in dendritic spines. (**a**) gSTED micrographs of surface expressed Ca_V_1.3_L_-HA (upper row and red in color overlay, confocal mode) co-labeled with eGFP-actin (green, gSTED) and synapsin (blue, gSTED). Ca_V_1.3_L_ clusters were found at the neck regions (a’), as well as at the base and within the head of dendritic spines (b’). (**b**) STORM microscopy of surface expressed Ca_V_1.3_L_-HA corroborates the findings of gSTED imaging and reveals that small, point-like channel clusters (c’, arrowheads) give rise to the anti-HA Ca_V_1.3_L_ fluorescence clusters typically observed in widefield microscopy (a’, arrowheads). a’, anti-HA, widefield; b’, eGFP-actin, widefield; c’, anti-HA STORM reconstruction; d’, color overlay: red, anti-HA widefield (HAw); green, eGFP-actin; white, anti-HA STORM reconstruction (HAs). Scale bars, 1 μm (**a**,**b**).

Because immunofluorescence analysis of Ca_V_1.3-HA suggested a localization of channel clusters in dendritic spines, we next employed super-resolution microscopy to determine the location of channel clusters within dendritic spines. gSTED microscopy revealed Ca_V_1.3-HA clusters within the neck region (Fig. 2a,a’), at the bases (Fig. 2a,b’), and within the spine heads of dendritic spines (Fig. 2a,b’). This finding was also corroborated by STORM microscopy (Fig. 2b) revealing that small, point-like channel clusters (arrowheads in Fig. 2b,c’) underlie the Ca_V_1.3_L_ fluorescence clusters, which are typically observed in widefield fluorescence microscopy. Larger and more intense Ca_V_1.3_L_ fluorescence spots can be formed by more than one underlying channel cluster (an example resolving two clusters is presented in Fig. 2b). Together this provides evidence that Ca_V_1.3 calcium channels are associated with dendritic spines in cultured hippocampal neurons.

### The C-terminal PDZ-binding sequence of Ca_V_1.3 promotes its neuronal membrane expression

**Figure 3:**
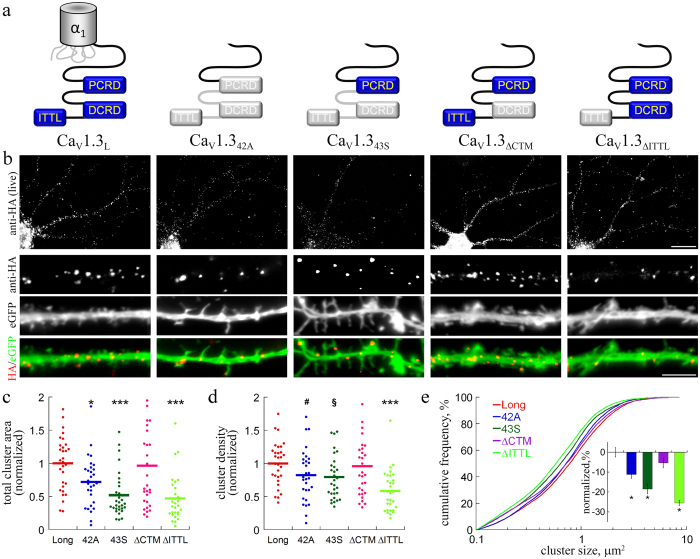
Membrane expression of extracellularly HA-tagged Ca_V_1.3 C-terminal splice variants and deletion mutants. (**a**) Schematic overview of the C-terminus of splice variants and mutated Ca_V_1.3. (**b**) anti-HA live cell staining of neurons co-transfected with HA-tagged Ca_V_1.3 splice variants or Ca_V_1.3_L_ mutants (anti-HA) and eGFP. Overall appearance of surface staining of the splice variants (Ca_V_1.3_42A_ and Ca_V_1.3_43S_) was similar to Ca_V_1.3_L_. Deletion of the DCRD (Ca_V_1.3_ΔCTM_) or the PDZ binding sequence ITTL (Ca_V_1.3_ΔITTL_) did not change the distribution pattern of channel clusters. (**c**) Quantitative analysis revealed that lack of the ITTL sequence (both in the short splice variants and in Ca_V_1.3_ΔITTL_) significantly reduced surface expression relative to Ca_V_1.3_L_ (data are normalized to the mean of Ca_V_1.3_L_). Both cluster density (**d**) and size ((**e**) Cumulative frequency distribution and fractional change) were reduced. Statistics: ANOVA with Holm-Sidak posthoc analysis (c: F_4,145_ = 8.8; p < 0.001; d: F_4,145_ = 5.8; p < 0.001; posthoc: *p < 0.05; ***p < 0.001; ^#^p = 0.13; ^§^p = 0.09). Kruskal-Wallis ANOVA with Dunn’s posthoc analysis (**e**: H_4_ = 77.4; p < 0,001; posthoc: *p < 0.05). Data from 2 culture preparations and 6 independent experiments, 30 cells and between 815 and 1291 channel clusters (E) were analyzed in each condition. Scale bars, 20 μm (overview) and 5 μm (dendritic segment).

In addition to the full-length Ca_V_1.3_L_ alternative splicing gives rise to two short (Ca_V_1.3_42A_, Ca_V_1.3_43S_) variants, which lack a major portion of the channels’ C-terminus and therefore differ with respect to the voltage-dependence of activation and calcium-dependent inactivation (refs 19 and 37; see schematic overview in Fig. 3a). In order to analyze the subcellular distribution of the short splice variants we generated HA-tagged channel constructs employing the same strategy as for Ca_V_1.3_L_. Interestingly, the dendritic area covered by channel clusters for Ca_V_1.3_42A_ and Ca_V_1.3_43S_ was reduced to 72% and 52%, respectively, when compared to the long isoform (Fig. 3b,c). This reduction in surface expression was due to a significantly decreased channel cluster size (Fig. 3e) and, to a smaller degree, reduced cluster density on the neuronal surface (Fig. 3d). Together this suggests a role of the Ca_V_1.3 C-terminus in the regulation of Ca_V_1.3 surface expression in hippocampal neurons.

Both short splice variants lack two previously identified important interaction sites. First, the distal C-terminal regulatory domain (DCRD), which forms a C-terminal modulator and is important for fine tuning of the calcium-dependent channel inactivation by intra-C-terminal interactions (CTM; ref. 37); second, the PDZ binding sequence (amino acid sequence ITTL) at the very distal C-terminus. This interaction sequence has previously been shown to be required for the interaction with modulatory PDZ domain proteins^13^,^27^,^38^. Thus, in order to test the hypothesis that either the DCRD or the PDZ binding sequence are necessary for stable neuronal surface expression, we generated deletion mutants by replacing the CTM modulatory sequence in the DCRD (DEME sequence, aa position 2073–2076) as well as the ITTL sequence with a V5 epitope tag, yielding the channel constructs Ca_V_1.3_ΔCTM_ and Ca_V_1.3_ΔITTL_, respectively. When expressed in tsA-201 cells, Ca_V_1.3_ΔCTM_ displayed biophysical properties similar to that of the short splice variants^39^. Deletion of the Ca_V_1.3_L_ ITTL sequence prevents binding to the PDZ domains of densin^13^ and shank^27^. Interestingly, surface expression of Ca_V_1.3_ΔITTL_ was strongly reduced, both in terms of the cluster density as well as cluster size, while the surface distribution of Ca_V_1.3_ΔCTM_ was not different from that of Ca_V_1.3_L_ (Fig. 3c–e). Thus, deletion of the PDZ ligand fully recapitulated the reduced surface expression observed in the short Ca_V_1.3 splice variants. Together these experiments demonstrate that the C-terminal PDZ-binding sequence of Ca_V_1.3 is critically involved in the regulation of Ca_V_1.3 membrane localization in dendrites.

### Expression of short Ca_V_1.3 splice variants induces dendritic spine elongation

**Figure 4:**
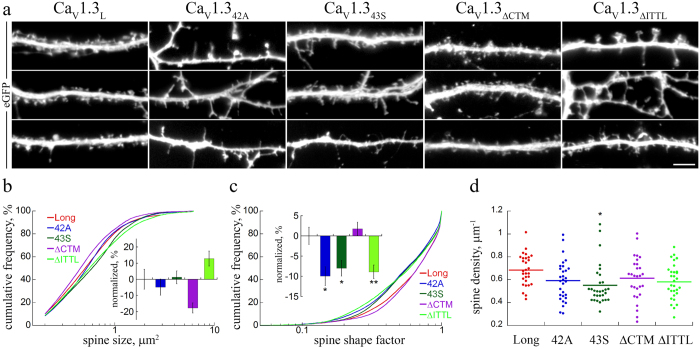
Expression of extracellularly HA-tagged Ca_V_1.3 C-terminal splice variants lacking ITTL alters dendritic spine morphology. (**a**) eGFP-labeled hippocampal neurons transfected with Ca_V_1.3_L_, Ca_V_1.3_42A_ and Ca_V_1.3_43S_, or the C-terminal ΔCTM and ΔITTL mutants (Ca_V_1.3_ΔCTM_ and Ca_V_1.3_ΔITTL_). Neurons expressing Ca_V_1.3_42A_, Ca_V_1.3_43S_, or Ca_V_1.3_ΔITTL_ showed a higher fraction of elongated, filopodia-like spines, in contrast to the stubby- and mushroom-like spines in neurons expressing Ca_V_1.3_L_ or Ca_V_1.3_ΔCTM_. (**b**–**d**) Quantitative analysis of dendritic spine size (**b**), spine shape factor (***c***), and spine density (**d**) upon expression of Ca_V_1.3_L_ (Long), Ca_V_1.3_42A_ (42A), Ca_V_1.3_43S_ (43S), Ca_V_1.3_ΔCTM_ (ΔCTM) and Ca_V_1.3_ΔITTL_ (ΔITTL). Statistics: b, Spine size: H_4_ = 26.8; p < 0.001 but no difference vs. control (Long); c, Shape factor: H_4_ = 32.2; p < 0.001; Kruskal-Wallis ANOVA with Dunn’s posthoc analysis; d, Spine density: F_4_ = 2.64; p = 0.036; *p < 0.05; Long vs. ITTL, p = 0.055, Long vs. 42A, p = 0.067, ANOVA with Holm-Sidak posthoc analysis. Data from 2 culture preparations and 6 independent experiments, between 320 and 683 dendritic spines from 30 cells were analyzed in each condition. Scale bar, 5 μm.

In heterologous expression systems the short splice variants Ca_V_1.3_42A_ and Ca_V_1.3_43S_ exhibit activation at a more negative voltage range as well as increased channel open probability^19^,^20^, both of which will result in increased calcium influx in fast spiking neurons. Because postsynaptic structure and function are critically controlled by the local concentration of calcium we next tested, whether the expression of the short splice variants affected the dendritic spine morphology. Indeed, when comparing the dendritic spine morphology of neurons expressing Ca_V_1.3_42A_ and Ca_V_1.3_43S_ with that expressing full-length Ca_V_1.3_L_ the overall structure of dendritic spines appeared less organized and, in particular, a larger fraction displayed an elongated, filopodia-like structure (Fig. 4a). In line with the effect on Ca_V_1.3 surface expression also deletion of the PDZ binding sequence (Ca_V_1.3_ΔITTL_) induced similarly elongated and partly enlarged dendritic spines. In contrast, neurons expressing Ca_V_1.3_ΔCTM_ developed predominantly mushroom-shaped dendritic spines similar to neurons expressing Ca_V_1.3_L_. A quantitative comparison of the dendritic spine parameters (Fig. 4b,c) between all tested Ca_V_1.3 splice variants and deletion mutants revealed a pronounced reduction of the spine shape factor in the short splices and the ΔITTL mutant when compared to full-length Ca_V_1.3_L_ or the ΔCTM mutant (Fig. 4c), but no significant effect on the spine size (Fig. 4b). Analysis of the spine shape factor reproduces values close to 1 for perfectly round objects and values close to zero for elongated objects. Thus the significant reduction of the spine shape factor indicates an elongation of the entire population of dendritic spines. In addition there was a tendency towards a reduced spine density in neurons expressing the short splices and the ΔITTL mutant (Fig. 4d).

### The PDZ domain proteins densin-180 and shank1b modulate the surface localization of the full-length Ca_V_1.3_L_

**Figure 5:**
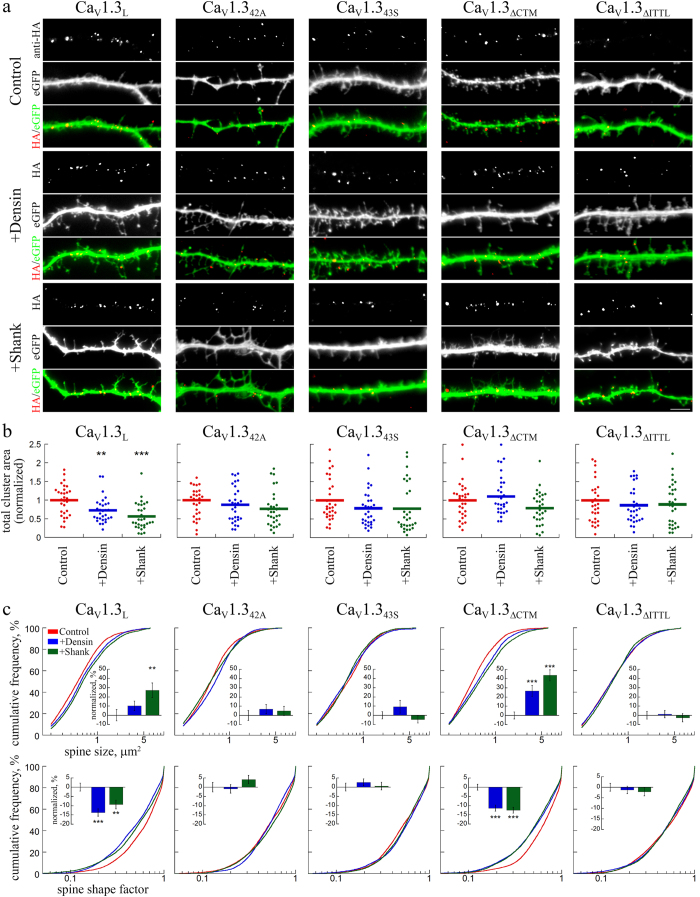
Co-expression of PDZ-domain proteins differentially affects membrane expression of extracellularly HA-tagged Ca_V_1.3 and alters dendritic spine morphology. (**a**) Live cell staining of neurons cotransfected with Ca_V_1.3-HA variants and densin-180 or shank1b together with eGFP. Co-expression of densin-180 or shank1b reduced total surface expression of Ca_V_1.3_L_ (see **b**) and induced the formation of more filopodia like dendritic spines, when expressed together with Ca_V_1.3_L_ and Ca_V_1.3_ΔCTM_. In contrast, densin and shank co-expression did not further affect the already elongated dendritic spines (cf. Fig. 4) in neurons expressing the short splice variants Ca_V_1.3_42A_, Ca_V_1.3_43S_, and Ca_V_1.3_ΔITTL_. (**b**) Quantification of total surface area, occupied by calcium channel clusters, normalized to the mean of control. Densin and shank significantly reduced membrane expression of the long splice variant Ca_V_1.3_L_. In the short splice variants and Ca_V_1.3_ΔITTL_ PDZ-domain proteins did not reduce total surface expression. Disruption of the C-terminal modulation (Ca_V_1.3_ΔCTM_) eliminated the densin-180-induced reduction in surface expression, but partly maintained the effect of shank1b (p = 0.11 vs control; p = 0.008 vs densin). (**c**) Quantitative analysis of morphological dendritic spines changes. Graphs show the cumulative frequency distribution of spines by size (upper panel) and shape factor (lower panel) as well as the fractional change (% difference to control, insets) induced by PDZ-domain proteins compared to the respective control condition (eGFP only). Statistics: **b**: ANOVA with Holm-Sidak posthoc analysis (Ca_V_1.3_L_: F_2,87_ = 10.6, p < 0.001; Ca_V_1.3_42A_: F_2,87_ = 1.7, p < 0.19; Ca_V_1.3_43S_: F_2,86_ = 1.4, p < 0.25; Ca_V_1.3_ΔCTM_: F_2,86_ = 3.7, p < 0.027; Ca_V_1.3_ΔITTL_: F_2,87_ = 0.44, p < 0.65; posthoc: **p < 0.01; ***p < 0.001). Data from 2 culture preparations, 6 independent experiments and 30 cells were analyzed in each condition. **c**: Kruskal-Wallis ANOVA with Dunn’s posthoc analysis (spine size: Ca_V_1.3_L_: H_2_ = 11.7, p = 0.003; Ca_V_1.3_42A_: H_2_ = 3.8, p = 0.15; Ca_V_1.3_43S_: H_2_ = 0.6, p = 0.73; Ca_V_1.3_ΔCTM_: H_2_ = 27.8, p < 0.001; Ca_V_1.3_ΔITTL_: H_2_ = 0.28, p = 0.87; spine shape factor: Ca_V_1.3_L_: H_2_ = 21.8, p < 0.001; Ca_V_1.3_42A_: H_2_ = 2.5, p = 0.29; Ca_V_1.3_43S_: H_2_ = 1.1, p = 0.57; Ca_V_1.3_ΔCTM_: H_2_ = 32.9, p < 0.001; Ca_V_1.3_ΔITTL_: H_2_ = 0.4, p = 0.80; posthoc: *p < 0.05). Scale bar, 5 μm.

Because the PDZ binding sequence of Ca_v_1.3 interacts with the postsynaptic PDZ domain proteins densin-180 and shank1b, we tested if these protein interactions regulate Ca_V_1.3 surface expression and dendritic spine stability. We co-expressed GFP-tagged densin-180^13^ or shank1b^27^ with the different Ca_V_1.3 splice variants and deletion mutants. When expressed in hippocampal neurons both GFP-tagged proteins, densin-180 and shank1b, displayed a striking clustered localization pattern in postsynaptic dendritic spines, as evidenced by the close apposition of the presynaptic marker synapsin with postsynaptic densin or shank1b clusters (Supplementary Fig. S2). Co-expression of either densin-180 or shank1b with full-length Ca_V_1.3_L_ significantly reduced surface localization by 27% and 43%, respectively, measured as the total cluster area occupied by Ca_V_1.3 channel clusters (Fig. 5a,b). This reduction of surface expression was not observed when densin-180 or shank1b were co-expressed with the short splice variants Ca_V_1.3_42A_ and Ca_V_1.3_43S_. Most importantly, densin-180 or shank1b also did not modulate the surface localization of the ΔITTL deletion mutant (Fig. 5b). Interestingly, co-expression of densin-180 and shank1b with Ca_V_1.3_ΔCTM_, the deletion mutant with an intact C-terminal PDZ binding sequence, did not affect surface expression to the same extent as with the long Ca_V_1.3_L_. Together these results demonstrate that PDZ domain proteins densin-180 and shank1b can modulate the surface localization of Ca_V_1.3_L_ and that this modulation critically depends on a functional PDZ binding sequence. The differential effects of shank1b and densin-180 co-expression on Ca_V_1.3_ΔCTM_ further suggest that modulation of surface expression by PDZ domain proteins may, in addition to the PDZ binding sequence, also require the integrity of the Ca_V_1.3 C-terminus.

### Co-expression of densin-180 and shank1b with full-length Ca_V_1.3 alters the morphology of dendritic spines

Both, densin-180 and shank1b, are components of the excitatory postsynaptic compartment and have previously been associated with the regulation of dendritic and postsynaptic morphology^22^. Importantly, both PDZ proteins can augment Ca_V_1.3 currents^13^,^28^. Because Ca_V_1.3 and the respective PDZ proteins are all located in dendritic spines, we reasoned that increasing levels of densin-180 or shank1b may act on postsynaptic Ca_V_1.3 channels and may thus contribute to the stability of dendritic spines. To test and quantify this hypothesis, we co-expressed GFP-tagged densin-180 and shank1b together with the different Ca_V_1.3 constructs in hippocampal neurons. Indeed, their co-expression together with Ca_V_1.3_L_ affected the morphology of dendritic spines (Fig. 5a) while expression of densin-180 and shank1b alone had no effect (Supplementary Fig. S3). Similar to the expression of the short splice variants or the ΔITTL mutant alone densin-180 and shank1b co-expression induced the formation of more elongated, partly filopodia-like shaped dendritic spines. This became mostly evident upon quantification of the dendritic spine size (Fig. 5c, upper panel) and the dendritic spine shape factor (Fig. 5c, lower panel). While co-expression of shank1b significantly increased spine size by 27%, densin-180 more strongly affected the spine shape, as revealed by a significant reduction of the spine shape factor (Fig. 5c, lower panel, insets) and a shift of the entire population of dendritic spines towards lower shape factor values in the cumulative frequency distribution plots (Fig. 5c, lower panel, Ca_V_1.3_L_).

As illustrated in Fig. 5a,c, densin-180 and shank1b effects critically depended on the presence of an intact ITTL-motif. Dendritic spine size and shape were not further affected by co-expression with the short splice variants Ca_V_1.3_42A_ and Ca_V_1.3_43S_ as well as with the ΔITTL deletion mutant. In contrast, spine sizes and shapes were altered when densin-180 and shank1b where co-expressed with the ΔCTM deletion mutant, in which the ITTL-motif was present (Fig. 5a,c).

**Figure 6:**
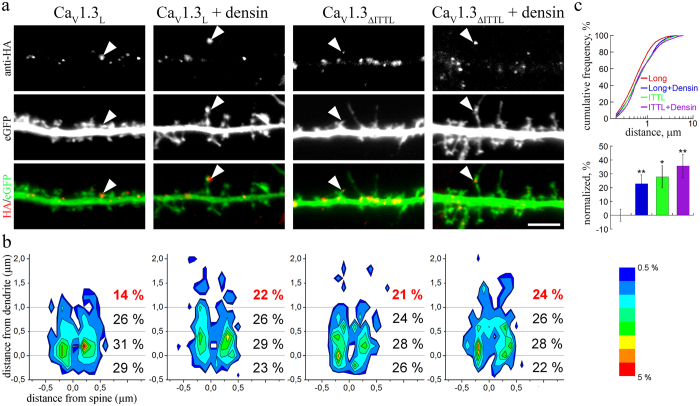
Redistribution of Ca_V_1.3 channel clusters in elongated dendritic spines. (**a**) Dendritic segments of hippocampal neurons (21 DIV) transfected with extracellularly HA-tagged Ca_V_1.3_L_ or Ca_V_1.3_ΔITTL_ without or with densin-180 (+densin). In conditions with elongated dendritic spines (Ca_V_1.3_L_ + densin-180, Ca_V_1.3_ΔITTL_, Ca_V_1.3_ΔITTL_ + densin-180) Ca_V_1.3 channel clusters are more often found within the neck region of dendritic spines and in the spine head (examples indicated by arrowheads). (**b**) Heatmap graphs showing the localization probability of Ca_V_1.3 channel clusters along the dendritic spine (y-axis, distance from dendrite) and laterally in relation to the main axis of the spine (x-axis, distance from spine). Negative y-values (below the 0 line) represent channel clusters positioned at the spine base. The fraction of channel clusters >1 μm from the spine base (red values) was significantly increased in the Ca_V_1.3_L_ + densin-180, Ca_V_1.3_ΔITTL_, and Ca_V_1.3_ΔITTL_ + densin-180 conditions (χ^2^ test, p = 0.04). (**c**) Cumulative frequency distribution of cluster distances from the spine base (upper panel; only positive values included) and fractional difference from the mean distance of control (Ca_V_1.3_L_) further demonstrate a redistribution of Ca_V_1.3 channel clusters along the elongated dendritic spines. Statistics: **b**: Chi-square test: χ^2^_(9)_ = 17.6, p = 0.04; **c**: ANOVA with Holm-Sidak posthoc analysis was performed on the log10-transformed raw data (F_3,978_ = 5.1, p = 0.002; posthoc: *p = 0.012, **p < 0.01). Scale bar, 5 μm.

Because Ca_V_1.3 channel clusters are located at the bases and within dendritic spines we next tested, whether dendritic spine elongation also affects the position of channel clusters. To this end we analyzed the position of extracellularly HA-tagged Ca_V_1.3_L_ channel clusters relative to the main axis of eGFP-filled dendritic spines in control neurons (Ca_V_1.3_L_) and conditions inducing elongation of spines (Ca_V_1.3_L_ + densin, Ca_V_1.3_ΔITTL_, and Ca_V_1.3_ΔITTL_ + densin, Fig. 6). In Fig. 6b the probabilities of HA-cluster localizations relative to the axis of the dendritic spines are plotted as probability heatmaps. As expected from the super-resolution imaging (Fig. 2) the majority of Ca_V_1.3_L_ channel clusters in the control condition were located below and lateral to the spine base (Fig. 6b), while only 14% of analyzed clusters were found at distances >1 μm from the spine base (distance form dendrite). In the transfection conditions inducing a higher proportion of elongated dendritic spine the fraction of Ca_V_1.3 clusters >1 μm was significantly increased to 21–24% (Fig. 6b). When plotting the cumulative frequency distribution of distances of Ca_V_1.3 clusters from the spine base (Fig. 6c, upper panel) and the difference of the mean distances to control it becomes evident that additionally also the entire population of Ca_V_1.3 clusters was shifted.

**Figure 7:**
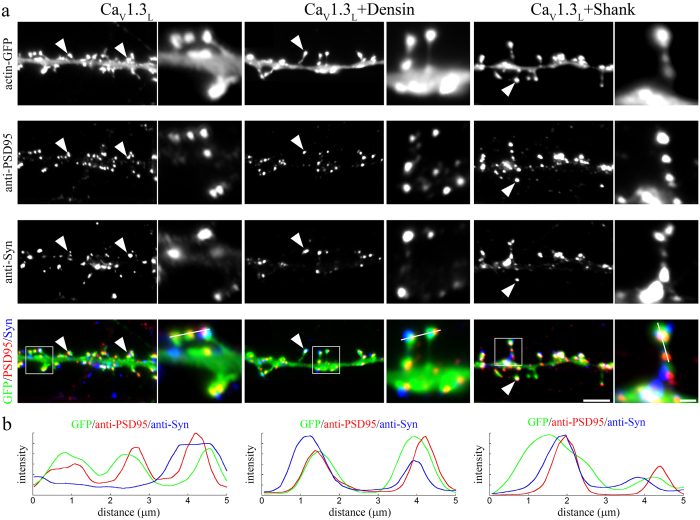
PDZ-domain proteins destabilize postsynaptic dendritic spines. (**a**) Localization of presynaptic synapsin-1 (anti-Syn) and postsynaptic PSD-95 (anti-PSD95) proteins on dendrites and spines of neurons expressing extracellularly HA-tagged Ca_V_1.3_L_ alone or together with densin-180 or shank1b. For visualization of dendritic spines actin-eGFP was co-expressed. In neurons expressing Ca_V_1.3_L_ alone PSD-95 immunolabeling in close apposition to synapsin is typically observed in the heads of mushroom like dendritic spines (arrowheads). In neurons co-expressing densin-180 or shank1b synaptic sites containing juxtaposed PSD-95 and synapsin are also observed within filopodia like dendritic spines (arrowheads). (**b**) Line scan analysis across triple-labeled dendritic spines indicates the co-localization of pre- and post-synaptic markers in the absence or presence of densin-180 or shank1b. Scale bars, 10 μm (overview) and 2 μm (magnified selections).

Finally, to test whether the observed dendritic spine alterations were due to a destabilization of postsynaptic dendritic spines or whether co-expression of densin-180 or shank1b induced the formation of new filopodia lacking postsynaptic specializations, we immunolabeled neurons co-expressing Ca_V_1.3_L_ and densin/shank with antibodies against synapsin as well as PSD-95 (Fig. 7). Although the morphology of dendritic spines was strongly altered upon densin-180 or shank1b co-expression, spines in all conditions displayed a correct alignment of presynaptic synapsin and postsynaptic PSD-95 on dendritic spine heads and along filopodia-like spines. This suggests that co-expression of densin-180 or shank1b with Ca_V_1.3_L_ modulates the shape of functional dendritic spines rather than inducing the excessive formation of new filopodia. Taken together our results also suggest that, compared to the short splice variants, the expression of full-length Ca_V_1.3_L_ stabilizes dendritic spines. Modulation by the interacting PDZ domain proteins induces similar consequences on the morphology of dendritic spines as expression of the functionally diverse short splice variants or expression of Ca_V_1.3_ΔITTL_. Thus, these experiments indicate that densin-180 and shank1b can modulate the morphology and stability of postsynaptic dendritic spines by their interaction with the C-terminus of Ca_V_1.3.

### Dendritic spine elongation correlates with increased Ca_V_1.3 current density in hippocampal neurons

**Figure 8:**
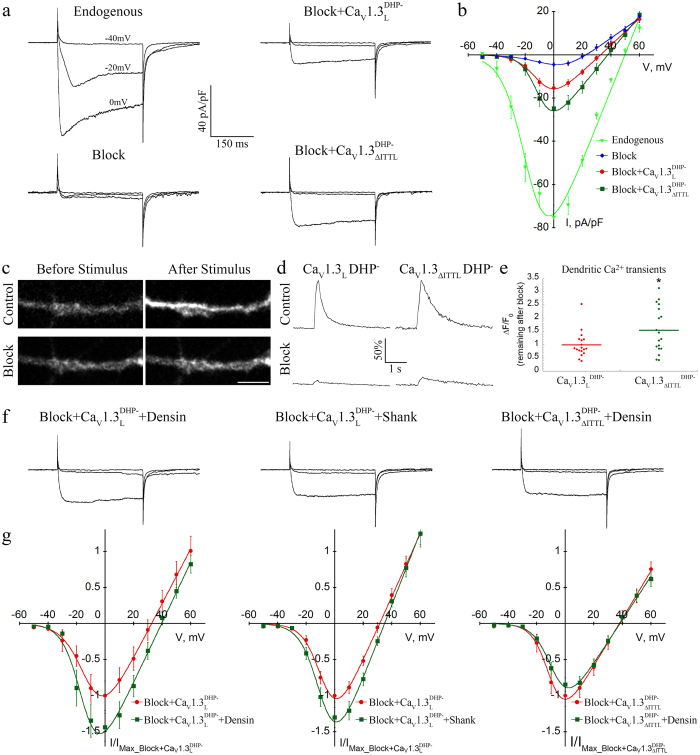
Current properties of Ca_V_1.3 channels expressed in cultured hippocampal neurons. (**a**,**b**) Representative Ba^2+^ whole-cell currents and I/V-curves recorded from untransfected hippocampal neurons with or without (Endogenous) channel blockers, and neurons expressing DHP insensitive Ca_V_1.3_L_^DHP-^ or Ca_V_1.3_ΔITTL_^DHP-^ without an extracellular HA-tag in the presence of channel blockers. (**c–e**) Measurements of dendritic calcium signals in response to 300 ms long depolarization steps (see Methods for details). (**c**) Micrograph of dendritic Fluo-4 fluorescence in DIV14 hippocampal neurons expressing DHP insensitive Ca_V_1.3_L_^DHP-^ channels before and after the depolarization stimuli. Upper panels (Control) show the increase in Fluo-4 fluorescence caused by calcium influx through all endogenous calcium channels. Lower panels (Block) show the modest increase in Fluo-4 intensity after the application of blockers for all endogenous calcium channels. Here the remaining calcium influx is mediated by Ca_V_1.3_L_^DHP-^ channels. (**d**) Time-course of the Ca^2+^ signal in dendrites (30 μm from the soma) of neurons expressing Ca_V_1.3_L_^DHP-^ or Ca_V_1.3_ΔITTL_^DHP-^ before (Control) and after application of the blocking cocktail (Block). Remaining Ca^2+^ signals were normalized to the amplitude of the control transient (100%). (**e**) Quantitative comparison of the remaining dendritic Fluo-4 Ca^2+^ signals after application of the blocking cocktail between Ca_V_1.3_L_^DHP-^ or Ca_V_1.3_ΔITTL_^DHP-^. ΔF/F_0_ after application of the blocking cocktail was normalized to the corresponding control value for each cell. Graph show data normalized to the mean (horizontal bar) of Ca_V_1.3_L_^DHP-^. Statistics: t = test, *p < 0.024. (**f**,**g**) Representative currents and I/V-curves (normalized to the peak current in cells transfected with Ca_V_1.3 alone) recorded in cells co-transfected with Ca_V_1.3_L_ and shank1b or densin-180, and Ca_V_1.3_ΔITTL_ with densin-180. PDZ-domain proteins increase current density only in the long splice variant of Ca_V_1.3, but not in the ΔITTL mutant. For statistics see text and Supplementary Tables S1 and S2. Scale bar, 5 μm.

In cultured hippocampal neurons dendritic spines are destabilized upon co-expressing densin-180 or shank1b with full length Ca_V_1.3_L_ as well as upon the sole expression of the short Ca_V_1.3 splice variants or the distal C-terminus deletion mutant ΔITTL. Because the short splice variants are activated at more negative voltages and show increased open probability we hypothesized that the observed effects on dendritic spine morphology may be induced by altered Ca_V_1.3 channel function. Unfortunately this hypothesis cannot be directly tested due to the lack of L-type calcium channel blockers specifically inhibiting endogenous Ca_V_1.2 or Ca_V_1.3 currents^40^. We therefore isolated Ca_V_1.3 currents in their native environment of hippocampal neurons, by introducing a point mutation into the Ca_V_1.3 cDNA (T1033Y), which has previously been shown to render the channel insensitive to dihydropyridine (DHP) L-type channel blockers (Ca_V_1.3_L_^DHP-^)^27^. Because L-type calcium channels contribute only partly to the total currents mediated by voltage-gated calcium channels in hippocampal neurons^41^,^42^ we first established a near-complete pharmacological block of all native calcium channels. To this end we used the combination of 800 nM ω-agatoxin IVA, 3 μM ω-conotoxin GVIA, 3 μM ω-conotoxin MVIIC, 1 μM SNX-482, and 30 μM nifedipine. Applying this blocking cocktail on 14 DIV old cultured hippocampal neurons reduced the total endogenous peak current density by 95% (Supplementary Table S1 and Fig. 8a,b). In order to verify the reduced DHP sensitivity of Ca_V_1.3_L_^DHP-^ and Ca_V_1.3_ΔITTL_^DHP-^ under our experimental conditions (30 μM nifedipine), we expressed them in tsA-201 cells and quantified the remaining peak currents before and 30 s after nifedipine application, which was sufficient to reach steady-state inhibition (Supplementary Fig. S4). 30 μM nifedipine robustly blocked currents through Ca_V_1.3_L_ (12% remaining current) and Ca_V_1.2, which was included as control. As expected, currents through the DHP-insensitive channel constructs Ca_V_1.3_L_^DHP-^ and Ca_V_1.3_ΔITTL_^DHP^ were only reduced to 62% and 59%, respectively. This confirms the dramatically reduced DHP sensitivity of these constructs even at high DHP concentrations. Most importantly, this permitted separation of transfected channels from endogenous channels in our neurons.

Neurons expressing the full-length and DHP-insensitive Ca_V_1.3_L_^DHP-^ displayed a significant ~4-fold increase in current densities above background (full block). The current density of the heterologously expressed channel reached 16% of the total currents sensitive to the blocking cocktail and had a half-maximal voltage of activation essentially identical to values previously reported for heterologously expressed Ca_V_1.3_L_ (Supplementary Table S1)^37^. Most importantly, peak currents of the DHP-insensitive ΔITTL deletion mutant Ca_V_1.3_ΔITTL_^DHP-^ were 60% larger than Ca_V_1.3_L_^DHP-^ currents representing an 81% increase after subtracting the residual blocking cocktail-sensitive current (p = 0.042, Holm-Sidak posthoc analysis, see Supplementary Table S1). To test whether the increase in somatic Ca_V_1.3_ΔITTL_ currents also affects local dendritic calcium signals, we recorded calcium transients by measuring the Fluo-4 intensity in dendritic segments at a distance of 30 μm from the cell soma (Fig. 8c–e). Local calcium transients were strongly reduced after application of the blocking cocktail (Fig. 8c). However, the amplitude of the remaining Fluo-4 signals (Fig. 8d) were 55% higher in neurons expressing Ca_V_1.3_ΔITTL_^DHP-^ compared to Ca_V_1.3_L_^DHP-^. Thus, deletion of the ITTL sequence resulted in a similar increase of local calcium transients and somatic currents through Ca_V_1.3 channels.

We next tested, whether co-expression of densin-180 or shank1b affected the current properties of Ca_V_1.3_L_^DHP-^. Compared to control (co-expression of eGFP) mean peak current densities of Ca_V_1.3_L_^DHP-^ upon co-expression of densin-180 were increased by 47% (63% after subtracting the residual blocking sensitive current; p = 0.028; t-test, n = 25 and 10; Fig. 8d, Supplementary Table S2). Co-expression of shank1b together with Ca_V_1.3_L_^DHP-^ induced a small increase in peak current densities by 19% (26% after subtracting the residual blocking sensitive current), which did approach significance (p = 0.07; t-test, n = 25 and 16; Fig. 8f,g, Supplementary Table S2). Most importantly, co-expression of densin-180 with the deletion mutant Ca_V_1.3_ΔITTL_^DHP-^ did not further increase the peak current density (p = 0.41; t-test, n = 12 each; Fig. 8f,g, Supplementary Table S2). Taken together, the increase in peak current densities mediated by Ca_V_1.3_ΔITTL_^DHP-^ as well as of currents mediated by Ca_V_1.3_L_^DHP-^ upon PDZ-protein co-expression is consistent with a role of increased Ca_V_1.3 currents in the dendritic spines elongation.

## Discussion

The experiments described here demonstrate for the first time an important role of Ca_V_1.3 for the stability of dendritic spines in hippocampal neurons and describe the cellular mechanism. We show that the intact C-terminal tail of the channel is required for the channel surface localization and for morphologically stable dendritic spines. Both features are subject to regulation by the expression of short C-terminal splice variants or an increased expression of densin-180 and shank1b. Modulation by densin-180 and shank1b requires the C-terminal ITTL sequence but not the CTM, which has previously been shown to be a critical regulator of calmodulin interaction with the channel. Our data suggest that enhanced Ca_V_1.3 currents result from an imbalanced expression of PDZ domain proteins or Ca_V_1.3 splice variants. These mechanisms may represent the critical step for changes in spine shape associated with synaptic plasticity and neurological disease.

Despite the fact that Ca_V_1.3 calcium channels are one of the two L-type calcium channels expressed in the central nervous system^10^,^43^,^44^ elucidating the precise subcellular localization of Ca_V_1.3 channels has been limited by a lack of high-quality antibodies. Therefore, here we employed high-resolution imaging of Ca_V_1.3 channels with an extracellularly exposed HA epitope. This approach was previously established for trafficking studies of the Ca_V_1.2 calcium channels^11^,^36^,^45^ and has subsequently been applied to Ca_V_1.3 channels^13^,^35^. Whereas previous experiments on the localization of Ca_V_1.3 were based on analyses of a mutant, not naturally occurring rat isoform^39^, we analyzed the localization of the full length human Ca_V_1.3 isoform (Ca_V_1.3_L_) using super-resolution microscopy. We find that Ca_V_1.3 channels, clustered on the base, neck, and head regions of dendritic spines, are ideally situated to contribute to the local calcium signal. Remarkably, the density of Ca_V_1.3 clusters and the number of channels within the clusters (cluster intensity) is considerably lower when compared with that of Ca_V_1.2, which is located at similar positions (Fig. 1) and was previously estimated to contain on average 8 channels per cluster^11^. However, the particular biophysical properties of Ca_V_1.3 channels, namely their activation at low voltages^15^,^16^, makes Ca_V_1.3 more sensitive to depolarization of the postsynaptic compartment.

The observed channel clustering could be attributable to two distinct mechanisms: either channels are clustered by diffusion traps, for example by the binding to postsynaptic scaffolding protein as has been shown for densin-180 and Ca_V_1.3^13^, or by a homo-oligomerization of the channels themselves. The latter has recently been suggested to occur via the C-terminus in a calcium-dependent manner for cardiac Ca_V_1.2 channels^46^. Our data also strongly support a role of the C-terminus for channel clustering, as clusters of Ca_V_1.3 splice variants lacking major portions of the C-terminus were significantly smaller. Moreover, the deletion of the PDZ binding sequence (ΔITTL) resulted in a similar decrease (Fig. 3e), while cluster sizes were not affected after deleting the DCRD region forming the C-terminal modulator (ΔCTM) fine-tuning calmodulin regulation^47^. The distal PDZ binding sequence is also subject to modulation by densin-180 and shank1b. Together these findings support a role of diffusion traps for Ca_V_1.3 clustering. Interestingly, a recent study revealed that clustering of the short splice Ca_V_1.3_42A_ via C-terminus-to-C-terminus interactions induces facilitation of the channel^48^. This, together with our present data, suggests distinct clustering mechanisms of short and long splice variants, homo-multimerization and PDZ-protein mediated clustering, respectively. Remarkably, formation of stable surface clusters of brain Ca_V_1.2 L-type channels is independent of postsynaptic scaffold proteins^49^ and channel clusters are maintained by a dynamic equilibrium of stable and mobile Ca_V_1.2 channels in the neuronal membrane^50^. Thus, clustering of the two distinct neuronal L-type channels Ca_V_1.3 and Ca_V_1.2 is regulated by fundamentally different mechanisms.

Densin-180, shank1b and Ca_V_1.3 have all previously been associated with altered dendritic spine morphology. Furthermore, both densin-180 and shank have been shown to augment currents through Ca_V_1.3 channels under certain physiological conditions. Densin-180, together with CaMKII, induces calcium-dependent facilitation of Ca_V_1.3^13^ and shank has been shown to mediate the G-protein-dependent inhibition of L-type currents in striatal medium spiny neurons by D2 dopaminergic and M1 muscarinic receptors^28^. Our study now provides compelling evidence that these effects converge on the regulation of Ca_V_1.3 and thus place Ca_V_1.3 channels at a critical position for regulating postsynaptic stability. The formation of filopodia-like dendritic spines is induced by the co-expression of densin-180 and shank1b together with Ca_V_1.3_L_. The same phenotype can be observed by expression of the short C-terminal Ca_V_1.3 splices alone, or by deletion of the C-terminal PDZ binding sequence. The observation that both experimental conditions affecting the structure of dendritic spines also correlated with increased Ca_V_1.3 current densities strongly point towards a major role of calcium entering via local Ca_V_1.3 channels. Using heterologous expression it has previously been established that short splice variants lack a CTM and therefore activate at more negative potentials than long isoforms, show enhanced coupling of voltage-sensor movement to pore-opening^20^, and display a higher current density and more pronounced calcium-dependent inactivation^19^,^37^. Ideally the physiological role of the distal C-terminus of Ca_V_1.3 should be addressed by recording endogenous Ca_V_1.3 currents in hippocampal neurons. However, at present it is not possible to pharmacologically separate native Ca_V_1.2 and Ca_V_1.3 channel currents^40^,^43^, or between the distinct Ca_V_1.3 splice variants^21^. We circumvented this problem by expressing DHP insensitive Ca_V_1.3 mutants and recorded Ba^2+^ currents while simultaneously blocking 95% of the endogenous currents. This homologous expression of recombinant Ca_V_1.3 channels in neurons reconstituted Ba^2+^ currents suitable for analyzing the effects of the C-terminal truncation and the co-expression of densin-180 and shank1b. To our knowledge this is the first time that Ca_V_1.3 currents have been isolated and recorded in their native neuronal environment. Similar to the current properties of heterologously expressed short splice variants^19^,^37^ current densities were increased in the ΔITTL mutant and upon densin-180 or shank1b co-expression. Yet, we did not observe the characteristic left shift in the voltage-dependence of activation found in the short splice variants. That left shift may be either attributable to the DCRD or, in consistence with a recent report^51^, may be less pronounced within the native cellular environment. Therefore our data suggest that the ITTL sequence is an important determinant of the current density, likely reflecting the single channel conductance, and may thus contribute to the previously described properties of the short splice variants.

Our study demonstrates that alternative splicing of Ca_V_1.3 channels and interactions with densin-180 and shank1b regulate dendritic spine morphology. Accumulating evidence strongly suggests that these specific interactions and modulations are indeed relevant for synaptic plasticity and neurological disease. First, morphological alteration of dendritic spines is a phenomenon commonly observed in classical LTP and LTD paradigms and neurological disorders (reviewed in ref. 22). The observed adaptations include new spine formation and spine enlargement in LTP, aberrant neuronal rewiring and hyperconnectivity in ASDs, reduced spine size and density in schizophrenia, spine loss in Alzheimer’s, and spine pruning in Parkinson’s disease^2^. Loss of spines in striatal medium spiny neurons is also associated with treatment-induced defects in connectivity and may thus underlie the development of L-DOPA induced dyskinesia^3^,^4^,^5^. Second, Ca_V_1.3 as well as densin-180 and shank1b have all been shown to be involved in the regulation of dendritic spines^22^. Third, densin-180 and shank1b proteins can modulate Ca_V_1.3 currents. Most importantly, all three proteins have been linked to neurological disorders. *SHANK* genes are likely causative for ASDs^33^, *CACNA1D* (Ca_V_1.3 α_1_-subunit) is an identified ASD risk gene^18^,^23^,^24^ and may be critical for loss of synapse stability in Parkinson’s disease^14^, and densin-180 knockout mice display symptoms related to schizophrenia and ASDs^34^. Finally, the effects on spine morphology and Ca_V_1.3 currents observed upon co-expression of densin-180 and shank1b are in line with a recently proposed model suggesting that *SHANK3* gene dosage affects brain function^33^.

## Conclusions

**Figure 9:**
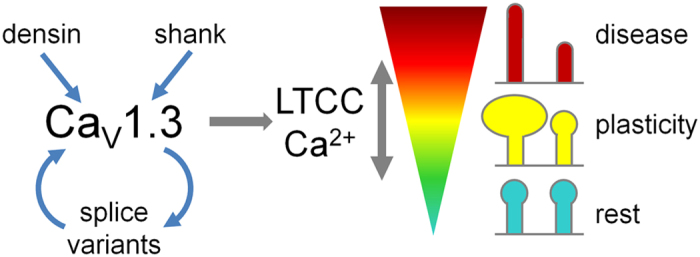
Model illustrating the bimodal function of Ca_V_1.3 channels in regulating dendritic spine morphology. Modulation of Ca_V_1.3 in dendritic spines by the postsynaptic scaffolding proteins densin-180 and shank1b or expression of different Ca_V_1.3 splice variants affects the local L-type calcium channel activity. Moderate or temporary increases in Ca_V_1.3 activity can induce synaptic plasticity (e.g. spine enlargement) in glutamatergic synapses. Altered Ca_V_1.3 channel activity, induced by a shift in splice variant expression or by increased local levels of postsynaptic PDZ-domain proteins, may result in aberrant spine morphology (elongation) and subsequent postsynaptic destabilization contributing to neurological disease.

Taken together our results demonstrate an important role of Ca_V_1.3 and its C-terminal interaction with densin-180 or shank1b in regulating postsynaptic dendritic spine morphology. Under physiological conditions this mechanism may be involved in the structural plasticity of glutamatergic synapses (Fig. 9). Conversely, altered regulation of Ca_V_1.3 channels by a shift in splice variants or imbalanced levels of postsynaptic PDZ-domain proteins may provide an important mechanism contributing to synaptic alterations associated with neurological disease.

## Methods

### Primary cultured hippocampal neurons

Low-density cultures of hippocampal neurons were prepared from 18-do embryonic BALB/c mice as described previously^11^,^50^,^52^,^53^. Briefly, dissected hippocampi were dissociated by trypsin treatment and trituration. For imaging experiments neurons were plated on poly-L-lysine-coated glass coverslips in 60 mm culture dishes at a density of ~3500 cells/cm^2^. After plating, cells were allowed to attach for 3–4 h before transferring the coverslips neuron-side down into a 60 mm culture dish with a glial feeder layer. For electrophysiology neurons were plated on a glial feeder layer. Neurons and glial feeder layer were maintained in serum-free neurobasal medium (Invitrogen) supplemented with Glutamax and B-27 supplements (Invitrogen). Ara-C (5 μM) was added 3 d after plating and, once a week, 1/3 of the medium was removed and replaced with fresh maintenance medium. Mice were bred and maintained at the central laboratory animal facility of the Medical University Innsbruck according to national and EU regulations and conforming to the Austrian guidelines on animal welfare and experimentation. The number of animals used to obtain cells for this project was annually reported to the Austrian Science ministry (bmwfw).

### Transfection of hippocampal neurons

Expression plasmids were introduced into neurons at 6 days *in vitro* (DIV) using Lipofectamine 2000-mediated transfection (Invitrogen) as described previously^11^. For co-transfection experiments (pβA-eGFP plus pβA-Ca_V_1.3-HA, pβA-eGFP plus pβA-Ca_V_1.3-HA plus either pEGFP-densin-180 or pGW1-GFP-shank1b) 1.5 μg or 2.5 μg of total DNA were used at a molar ratio of 1:2 or 1:2:2, respectively. Cells were processed for patch clamp experiments and immunostaining 7–8 and 12–13 days, respectively, after transfection.

### Expression vectors and cloning procedure

To facilitate neuronal expression all constructs were cloned into a eukaryotic expression plasmid containing a neuronal chicken β-actin promoter (pβA)^11^,^54^, derived from pβA-eGFP-γ-cytoplasmic-actin^54^.

### pβA-Ca_V_1.3_L_

Ca_V_1.3_L_ cDNA (Genbank accession number EU363339) was isolated from the peGFP^+^ vector^16^ and cloned into the pβA expression vector in the following way [nucleotide numbers (nt) are given in parentheses and asterisks indicate restriction sites introduced by polymerase chain reaction (PCR)]: The PCR-generated HindIII^*^/SalI^*^–SpeI fragment of Ca_V_1.3_L_ (nt 1–747), with the Kozak sequence (CCTACC) before the starting codon, was ligated into the HindIII/SpeI-cleaved plasmid pβA-PL^36^, yielding the subclone pβA-Ca_V_1.3 (nt 1–747). The Ca_V_1.3_L_ fragment SpeI–HpaI (nt 748–6414) was ligated into the corresponding sites of pβA-Ca_V_1.3 (nt 1–747) yielding pβA-Ca_V_1.3_L_.

### pβA-Ca_V_1.3_L_-HA

The hemagglutinin (HA) tag was inserted by SOE-PCR into the extracellular loop connecting IIS5-IIS6 according to a similar strategy used for rat Ca_V_1.3-HA^13^ and Ca_V_1.2-HA^45^. Briefly the cDNA sequence of Ca_V_1.3 (nt 1683–3096) was PCR amplified with overlapping primers introducing the HA tag in separate PCR reactions using pβA-Ca_V_1.3_L_ as template. The two separate PCR products were then used as templates for a final PCR reaction with flanking primers to connect the nucleotide sequences. This fragment was then BsrGI/PmlI digested and cloned into the respective sites of pβA-Ca_V_1.3_L_ yielding pβA-Ca_V_1.3_L_-HA.

### pβA-Ca_V_1.3_42A_-HA and pβA-Ca_V_1.3_43S_-HA

To generate the HA-tagged Ca_V_1.3_42A_ and Ca_V_1.3_43S_ constructs, we cloned the respective spliced cDNA fragments into the backbone of the already HA-tagged pβA-Ca_V_1.3_L_-HA. To this end the cDNA of the C-terminal regions or Ca_V_1.3_42A_ and Ca_V_1.3_43S_, which contain the respective splice sequences, were isolated from peGFP^+^-Ca_V_1.3_42A_^37^ and peGFP^+^-Ca_V_1.3_43s_^19^, respectively, by BstEII/AvrII digestion. The two fragments were then ligated into the respective sites of pβA-Ca_V_1.3_L_-HA, yielding pβA-Ca_V_1.3_42A_-HA and pβA-Ca_V_1.3_43S_-HA.

### pβA-Ca_V_1.3_L_-HA-ΔCTM

The C-terminal gating modulator domain, consisting of the DEME amino acids (aa position 2073–2076), was deleted and a V5 epitope was introduced in the same position by SOE-PCR. Briefly the cDNA sequence of Ca_V_1.3 (nt 5882–7320) was PCR amplified with overlapping primers introducing a V5 tag and deleting the CTM domain in separate PCR reactions using pβA-Ca_V_1.3_L_-HA as template. The two separate PCR products were then used as templates for a final PCR reaction with flanking primers to connect the nucleotide sequences. This fragment was then AvrII/EcoRV digested and co-ligated with BsrGI/AvrII and BsrGI/EcoRV fragments of pβA-Ca_V_1.3_L_-HA, yielding pβA-Ca_V_1.3_L_-HA-ΔCTM.

### pβA-Ca_V_1.3_L_-HA-ΔITTL

The last 4 C-terminal amino acids (ITTL, aa position 2134–2137) were deleted and a V5 epitope was introduced in the same position by SOE-PCR. Briefly the cDNA sequence of Ca_V_1.3 (nt 5888–7320) was PCR amplified with overlapping primers introducing the V5 tag and deleting the ITTL residues in separate PCR reactions using pβA-Ca_V_1.3_L_-HA as template. The two separate PCR products were then used as templates for a final PCR reaction with flanking primers to connect the nucleotide sequences. This fragment was then AvrII/ApaI digested and co-ligated with BstEII/AvrII and BstEII/ApaI fragments of pβA-Ca_V_1.3_L_-HA, yielding pβA-Ca_V_1.3_L_-HA-ΔITTL.

### pβA-Ca_V_1.3_L_-T1033Y(DHP-)

The T1033Y mutation was introduced by SOE-PCR. Briefly the cDNA sequence of Ca_V_1.3 (nt 2916–4851) was PCR amplified with overlapping primers introducing the mutation in separate PCR reactions using pβA-Ca_V_1.3_L_ as template. The two separate PCR products were then used as templates for a final PCR reaction with flanking primers to connect the nucleotide sequences. This fragment was then PmlI/BstEII digested and ligated into the corresponding sites of pβA-Ca_V_1.3_L_, yielding pβA-Ca_V_1.3_L_-T1033Y(DHP-).

### pβA-Ca_V_1.3_L_-ΔITTL-T1033Y(DHP-)

A fragment (nt 1–3103) containing the T1033Y mutation was isolated from pβA-Ca_V_1.3_L_-T1033Y(DHP-) by PmlI/PacI digestion and ligated into the corresponding sites of pβA-Ca_V_1.3_L_-HA-ΔITTL, yielding pβA-CaV1.3_L_-ΔITTL-T1033Y(DHP-).

Sequence integrity of all newly generated constructs was confirmed by sequencing (MWG Biotech, Martinsried, Germany).

### Antibodies

Primary antibodies used were as follows: rat anti-HA 1:100 (clone 3F10), mouse anti-GFP (clones 7.1 and 13.1) (Roche Diagnostics); mouse anti-Synapsin 1:2000 (clone 46.1), rabbit anti-PSD-95 1:1000 (Synaptic Systems, Goettingen, Germany). Secondary antibodies used were as follows: goat anti-rat Alexa 594 (1:4,000) and anti-rat Alexa 647 (1:400), goat anti-mouse Alexa 488 (1:2,000), and Alexa 350 (1:500); goat anti-rabbit Alexa 594 (1:4,000) (all from Invitrogen); goat anti-mouse Abberior STAR 440SX (Abberior GmbH, Göttingen, Germany).

### Immunocytochemistry and microscopy

For surface staining Ca_V_1.3-HA transfected neurons were incubated with anti-HA for 20 min at 37 °C. Coverslips were rinsed in HBSS and fixed with 4% paraformaldehyde for 10 min. After fixation, neurons were washed with PBS for 30 min, blocked with 5% goat serum for 30 min, and labeled with anti-rat Alexa Fluor 594 (1:4000, 1 h). Coverslips were mounted in p-phenylenediamine glycerol to retard photobleaching^55^ and observed with an Axio Imager microscope (Carl Zeiss) using 63×, 1.4 NA oil-immersion objective lens. Images were recorded with a cooled CCD camera (SPOT Imaging Solutions, Sterling Heights, MI USA). Permeabilized staining was performed as described previously^36^. For super-resolution microscopy hippocampal neurons were co-transfected with pβA-Ca_V_1.3_L_-HA and pβA-eGFP-γ-cytoplasmic-actin and live-cell immunostained with anti-HA as described. For STORM microscopy neurons were labeled with the secondary anti-rat Alexa Fluor 647 (1 h) antibody. Coverslips were mounted in a mixture of 10% (v/v) Vectashield (Vector Laboratories LTD, Peterborough, UK) with 90% glycerol buffer (5% Tris-HCl/95% glycerol) and imaged on an iMIC microscope (TILL photonics) equipped with an OrcaFlash 4.0 CMOS camera (Hamamatsu Photonics Deutschland GmbH) using a 60×, 1.3 NA oil-immersion objective lens. STORM images were reconstructed using rapidSTORM 3 software^56^. For gSTED microscopy neurons with primary live-cell applied anti-HA antibodies were labeled with the secondary anti-rat Alexa Fluor 594 (1:4000, 1 h). Subsequently, neurons were fixed again in 4% paraformaldehyde for 5 min, washed, permeabilized and blocked again with 5% goat serum for 30 min. Neurons were then labeled with the mouse anti-Synapsin antibody overnight at 4° and finally incubated with the secondary anti-mouse Abberior STAR 440SX antibody (1:100). Coverslips were mounted in DABCO/Glycerol to retard photobleaching. Neurons were imaged on a Leica TCS SP8 gSTED microscope equipped with a HCX PL APO 100x/1.40 OIL objective (Leica microsystems GmbH, Germany). Fluorophores were recorded in the following sequence (excitation/detection range wavelength in nm): Alexa 594 (Ca_V_1.3_L_-HA; 598 + 606/610-701; gate 0.5–6 ns in confocal mode), eGFP (eGFP-γ-cytoplasmic-actin, 505 + 513/519–580; gate 1–6 ns, STED laser at 592 nm), and STAR440 (m-Synapsin; 470/476-576; gate 0.8–6 ns, STED laser at 592). Raw images were channel dye separated (LAS AF software, Leica microsystems GmbH, Germany) and deconvolved using Huygens Professional software (Scientific Volume Imaging, Hilversum, The Netherlands).

### Quantification of dendritic spines and Ca_V_1.3-HA clusters

Fourteen-bit gray scale images of anti-HA (red channel) and eGFP (green channel) were acquired and analyzed as described previously^49^,^50^. Briefly, corresponding images were aligned and the eGFP image was used to select the regions of interest (ROIs) for measuring the numbers of Ca_V_1.3-HA clusters, area occupied by Ca_V_1.3-HA clusters and spine parameters with MetaMorph software (Molecular Devices, Sunnyvale CA, USA). The spine shape factor was calculated as 4πA/P^2^, where A is the area of the object and P is its perimeter. A value of 0 indicates an linear object, whereas a value of 1 indicates a circle. The positions of the Ca_V_1.3-HA clusters along dendritic spines were analyzed using a custom programmed MetaMorph journal. Briefly, for each neuron a segment of a dendritic shaft was selected, the background was flattened and the Ca_V_1.3-HA images were thresholded. In each dendritic spine containing an HA-cluster the spine extension (from spine base to spine tip) and the HA-cluster position (from spine base to HA-cluster centroid) was recorded.

### Electrophysiology

Barium currents through Ca_V_1.3^DHP-^ channels were recorded using the whole-cell patch-clamp technique. Patch pipettes were pulled from borosilicate glass (Harvard Apparatus), fire-polished (Microforge MF-830, Narishige), and had resistances of 2.5–4 MΩ when filled with the following (in mM): 120 cesium methanesulfonate, 1 MgCl_2_, 0.1 CaCl_2_, 10 HEPES, 0.5 EGTA, 2 Mg-ATP, 0.3 a-GTP (pH 7.2 with CsOH). The bath solution contained the following (in mM): 10 BaCl_2_, 110 NaCl, 20 TEA-Cl, 5 4-aminopyridine (4-AP), 10 HEPES, 2 MgCl_2_, 3 KCl, 10 glucose, 0.001 TTX (pH 7.4 with NaOH). Currents were recorded with an EPC 10 amplifier controlled by PatchMaster software (HEKA Elektronik Dr. Schulze GmbH, Germany). Linear leak and capacitive currents were digitally subtracted with a P/4 prepulse protocol. The current–voltage dependence was fitted according to equation 1:


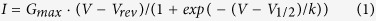


where G_max_ is the maximum conductance of the Ca_V_1.3 calcium channels, V_rev_ is the extrapolated reversal potential of the calcium current, V_1/2_ is the potential for half-maximal conductance, and k is the slope. Neurons were held at −50 mV to inactivate currents through T-type calcium channels, all other endogenous calcium channels were blocked with the following channel inhibitors: 800 nM ω-agatoxin IVA, 3 μM ω -conotoxin GVIA, 3 μM ω -conotoxin MVIIC, 1 μM SNX-482 (all from Alomone labs, Jerusalem, Israel) and 30 μM nifedipine (Sigma-Aldrich, St. Louis, MO, USA).

### Fluorescent calcium measurements

Hippocampal neurons were loaded with the membrane permeable calcium-sensitive fluorescent dye Fluo-4 AM by incubation for 25 min at 37 °C in Tyrode solution supplemented with 5 μM Fluo-4 AM and 0.02% pluronic-127 detergent. At the end of the incubation cells were washed twice and then incubated in Tyrode solution for 20 min to complete cytoplasmic dye deesterification. Fluo-4 was excited at ~490 nm and emitted light was detected at ~525 nm using an Olympus IX71 microscope equipped with a 40×, 0.6 NA LUCPLFLN objective. Images were recorded with cooled SPOT Pursuit camera (SPOT Imaging Solutions, Sterling Heights, MI USA) and VisiView Imaging Software (Visitron Systems, GesmbH, Puchheim, Germany). Cells were stimulated with the patch pipette by a 300 ms depolarization pulse from −70 to 0 mV. After application of the blocking cocktail, calcium transients were quantified after 6 stimulation pulses (10 s inter-pulse intervall).

### Cell culture and transfection of tsA-201 cells

tsA-201 cells (human embryonic kidney cells expressing SV40 temperature sensitive T antigen, European Collection of Cell Culture, 96121229), were cultured in Dulbecco’s modified Eagle’s medium (DMEM), containing 10% fetal calf serum (Gibco, 10500.064), 2 mM glutamine (Sigma, G753), penicillin (10 units/ml, Sigma, P-3032) and streptomycin (10 μg/ml, Sigma, S-6501) and maintained at 37 °C in a humidified environment in the incubator with 5% CO_2_. After the cells reached 80% confluency they were split using 0.05% trypsin for cell dissociation. Cells were plated on 10-cm culture dishes one day prior to transfection. For whole-cell patch-clamp recordings, tsA-201 cells were transiently transfected with different α_1_-subunits (3 μg, Ca_v_1.3_L_, Ca_v_1.3_L_-HA, Ca_v_1.3^DHP-^, Ca_v_1.3_ΔITTL_^DHP-^ or Ca_v_1.2) together with auxiliary β_4b_ (2 μg) and α_2_δ-1 (2.5 μg, rabbit, NM_001082276) subunits using Ca^2+^-phosphate precipitation^40^. 24 h after transfection cells were replated onto a 35-mm culture dish coated with poly-L-lysine and cultured at 30 °C and 5% CO_2_. Cells were kept for 24–48 h till further usage.

### Electrophysiology of tsA-201 cells

For whole-cell patch-clamp recordings borosilicate glass electrodes (203-776-0664 Warner Instruments and 64–0792, Harvard Apparatus, USA) with a final resistance of 1.5–3.5 MΩ (tsA-201 cells) were pulled using a micropipette puller (Sutter instruments, P-97) and fire polished afterwards (Microforge, Narishinge MF-830). All recordings were performed at room temperature (21–23 °C) in whole-cell configuration using the Axopatch 200B amplifier (Axon instruments), digitized at 50 kHz (Digitizer 1322A, Axon instruments), low-pass filtered at 5 kHz and compensated for 60–90% of the series resistance. The recording solutions contained in mM: *bath*: 15 BaCl_2_, 10 HEPES, 150 choline-Cl and 1 MgCl_2_, adjusted to pH 7.4 with CsOH; *intracellular*: 135 CsCl, 10 HEPES, 10 Cs-EGTA, 1 MgCl_2_, 4 mM Na_2_ATP adjusted to pH 7.4 with CsOH. To determine the current-voltage (I-V) relationship, a 30 ms square pulse protocol to different voltages was applied. The holding potential was set to −80 mV and currents were leak subtracted using a P/4 protocol. Resulting I-V curves were fitted according to equation 1. The voltage dependence of Ca^2+^ conductance was fitted according to a Boltzman distribution (equation 2):


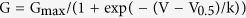


For analysis of current densities, only recordings from the same transfections were compared. To investigate the surface expression of Ca_v_1.3_L_ and Ca_v_1.3_L_-HA constructs, we analyzed the amplitude of the integrated ON-gating current at the reversal potential. For pharmacological experiments, cells were perfused by an air pressure-driven perfusion system (BPS-8 Valve Control System, ALA Scientific Instruments, flow rate: 250 μl/min). Bath solution containing 30 μM nifedipine was applied after at least four constant control sweeps during perfusion with bath solution only. To assess the effect of 30 μM nifedipine on different constructs, cells were depolarized from a holding potential of −50 mV to V_max_ for 100 ms at 0.1 Hz and peak currents before and 30 s after nifedipine application, which was sufficient to reach the equilibrium, were analyzed. Leak subtraction was performed offline.

### Statistical Analysis

Results are expressed as means ± S.E.M. Data were organized and analyzed using MS Excel, InStat (GraphPad Software, La Jolla, CA USA), Clampfit 10.2 (Axon Instruments), Graph Pad Prism 5.1 software (GraphPad Software Inc.), Sigmaplot 8 and 12 (Systat Software GmbH, Erkrath, Germany), and SPSS (SPSS Inc, Chicago IL, USA) statistical software. Statistical tests are mentioned in figure legends and text, data are presented as mean ± SEM except were otherwise noted.

## Additional Information

**How to cite this article**: Stanika, R. *et al*. Splice variants of the Ca_v_1.3 L-type calcium channel regulate dendritic spine morphology. *Sci. Rep.* **6**, 34528; doi: 10.1038/srep34528 (2016).

## Supplementary Material

Supplementary Information
