# Supplementary material for: Splice variants of the CaV1.3 L-type calcium channel regulate dendritic spine morphology
Source: Sci Rep. 2016 Oct 6;6:34528. doi: 10.1038/srep34528 (PMC5052568; doi:10.1038/srep34528)
Supplement: Supplementary Information [file srep34528-s1.pdf]

Supplementary Information

Splice variants of the $Ca_V1.3$ L-type calcium channel regulate dendritic spine morphology

by Ruslan Stanika, Marta Campiglio, Alexandra Pinggera, Amy Lee, Jörg Striessnig, Bernhard E. Flucher, and Gerald J. Obermair

Supplementary Tab. S1. Current properties of endogenous and residual (blocked) as well as $Ca_V1.3_L^{DHP-}$ and $Ca_V1.3_{\Delta ITTL}^{DHP-}$ calcium channels.

	Endogenous			Block		
	Mean	±SEM	n	Mean	±SEM	n
CD (pA/pF)	-75.3	5.8	14	-4.1	1.0	14
V_{50act}(mV)	-17.2	2.2	14	7.1	3.0	14
V_{rev} (mV)	50.7	1.2	14	21.6	3.9	14

	Block+$Ca_V1.3_L^{DHP-}$			Block+$Ca_V1.3_{\Delta ITTL}^{DHP-}$		
	Mean	±SEM	n	Mean	±SEM	n
CD (pA/pF)	-15.4	1.6	25	-24.6	3.8	12
V_{50act}(mV)	-7.2	1.6	25	-9.8	1.1	12
V_{rev} (mV)	33.6	2.1	25	37.5	3.0	12

CD, current density; V_{50act}, half-maximal voltage of activation; V_{rev}, reversal potential. Statistics for CD: ANOVA: $F_{(3, 61)}$=90.6; p<0.001; Holm-Sidak posthoc analysis: Endogenous vs Block, p<0.001; Block vs Block+$Ca_V1.3_L^{DHP-}$, p=0.019; Block+$Ca_V1.3_L^{DHP-}$ vs Block+$Ca_V1.3_{\Delta ITTL}^{DHP-}$, p=0.042.

Supplementary Tab. S2. Current properties of $Ca_V1.3_L^{DHP-}$ and $Ca_V1.3_{\Delta ITTL}^{DHP-}$ co-expressed with densin-180 and shank1b.

		Block+$Ca_V1.3_L^{DHP-}$			Block+$Ca_V1.3_{\Delta ITTL}^{DHP-}$		
		Mean	±SEM	n	Mean	±SEM	n
CD (pA/pF)	Control	-15.4	1.6	25	-24.6	3.8	12
	+Densin	-23.3	3.0	10	-20.7	2.4	12
	+Shank	-17.8	1.6	16	-	-	-
V_{50act}(mV)	Control	-7.2	1.5	25	-9.8	1.1	12
	+Densin	-13.1	1.8	10	-6.9	0.9	12
	+Shank	-7.0	0.9	16	-	-	-
V_{rev} (mV)	Control	33.6	2.1	25	37.5	3	12
	+Densin	39.2	2.6	10	39.1	2.0	12
	+Shank	35.8	1.4	16	-	-	-

CD, current density; V_{50act}, half-maximal voltage of activation; V_{rev}, reversal potential. For statistics, which was performed on the data normalized to the respective control experiments, see text.

Supplementary Figure S1. The extracellular HA-tag does not affect current properties and surface expression of Ca$_V$1.3$_L$

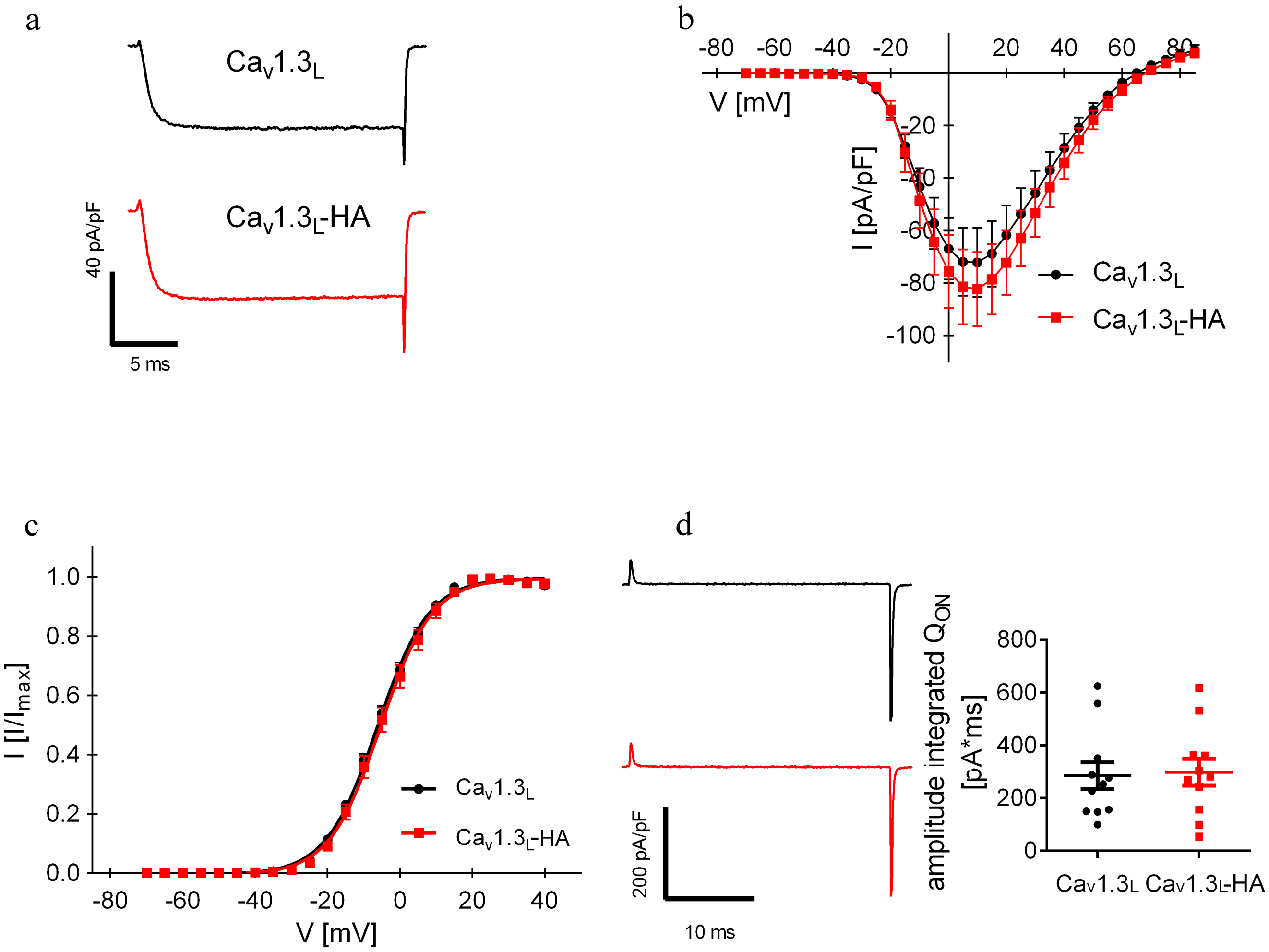

Current properties of Ca$_V$1.3$_L$ and Ca$_V$1.3$_L$-HA channels expressed in tsA-201 cells. ***a***,***b***, Representative Ba^{2+} whole-cell currents (***a***) and I/V-curves (***b***) recorded from tsA-201 cells transfected with the respective Ca$_V$1.3$_L$ construct plus β_{4b}, $\alpha_2\delta$-1, and eGFP are indistinguishable for wildtype (n=16) and HA-tagged (n=15) Ca$_V$1.3$_L$. ***c***, Identical steady-state activation curves for Ca$_V$1.3$_L$ and Ca$_V$1.3$_L$-HA show no effect of the HA-tag on the voltage-dependence of activation. ***d***, Representative ON-gating charges (left panel) and amplitudes of integrated ON-gating charges (Q_{ON}, right panel) recorded at the reversal potential for Ca$_V$1.3$_L$ and Ca$_V$1.3$_L$-HA (n=11 for both). Horizontal bars represent means ± SEM. Data from three independent transfections. Statistics: unpaired Student's t-test (b, peak current density, p=0.559; c, V_{50act}, p=0.405; d, amplitudes of integrated Q_{ON}, p=0.618).

Supplementary Figure S2. Postsynaptic localization of the GFP-fusion proteins densin-180 and shank1b.

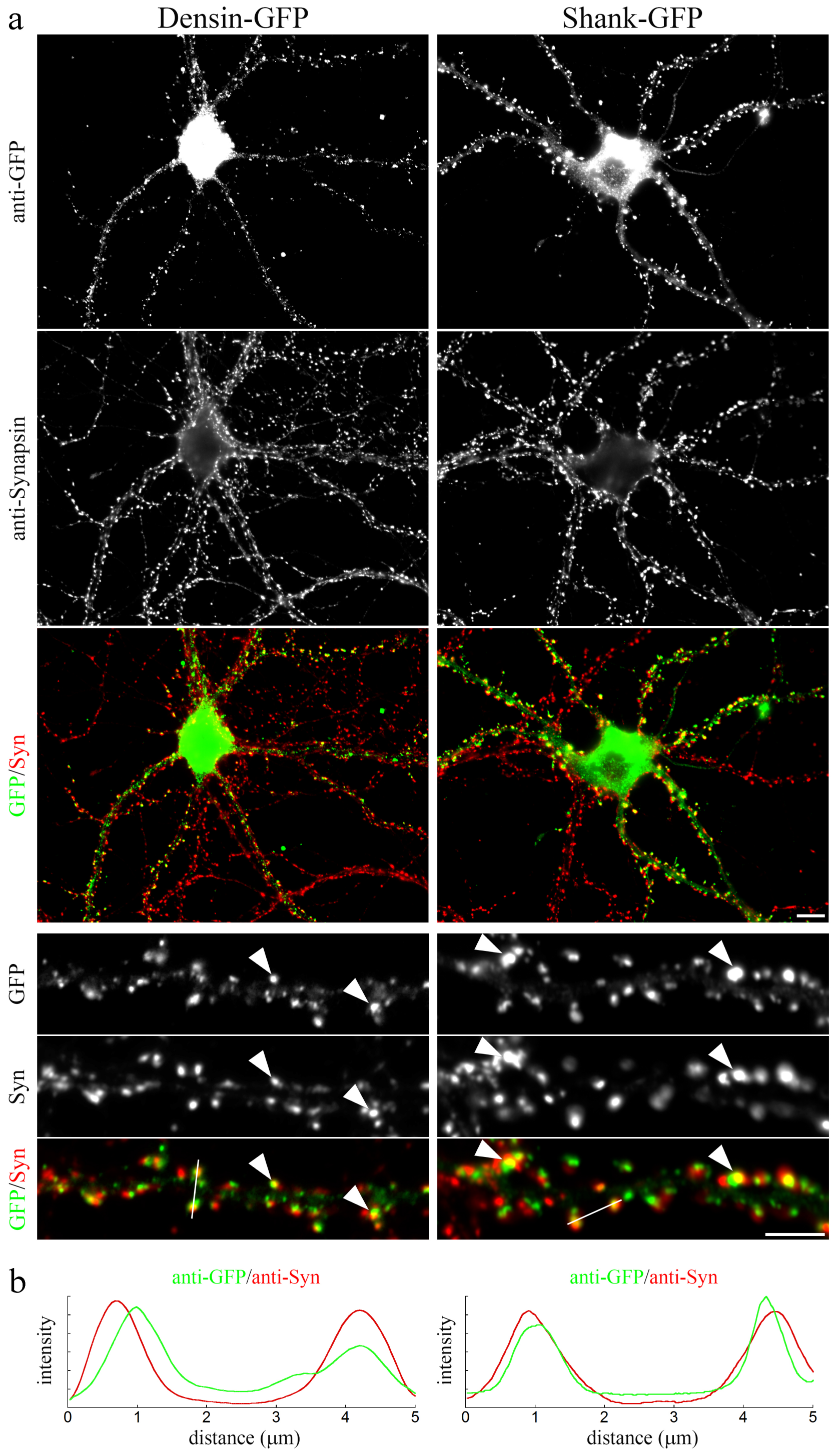

a, Hippocampal neurons were transfected with densin-180-GFP or GFP-shank1b and labeled with anti-GFP and anti-synapsin-1. Both, densin and shank, show a punctate localization pattern along the dendrites. The close-apposition of presynaptic boutons labeled with synapsin in magnified dendritic segments shows the postsynaptic localization of these two PDZ domain proteins (examples indicated by arrowheads). ***b***, Line scan analyses further corroborates the apposition of postsynaptic densin-GFP and GFP-shank1b with the presynaptic synapsin. Scale bars, 10 µm (overview) and 5 µm.

Supplementary Figure S3. Expression of PDZ-domain proteins densin-180 and shank1b in hippocampal neurons does not alter dendritic spine morphology

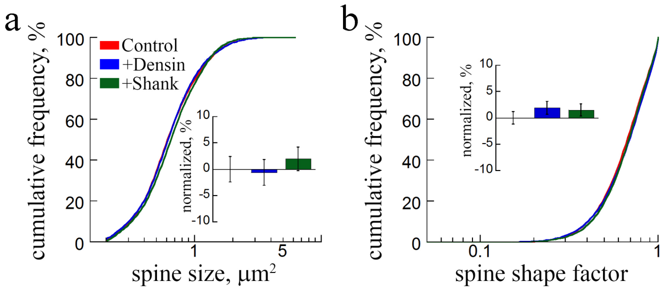

Quantitative analysis of morphological dendritic spines parameters upon expression of densin-180 or shank1b in cultured hippocampal neurons. Graphs show the cumulative frequency distribution of spines by size (***a***) and shape factor (***b***) as well as the fractional change (% difference to control, insets) induced by PDZ-domain proteins compared to the control condition (eGFP only). Kruskal-Wallis ANOVA, spine size: H_2=3.56, p=0.169; spine shape factor: H_2=1.57, p=0.456.

Supplementary Figure S4. Strongly reduced dihydropyridine sensitivity in DHP-insensitive $Ca_V1.3_L^{DHP-}$ and $Ca_V1.3_{\Delta ITTL}^{DHP-}$

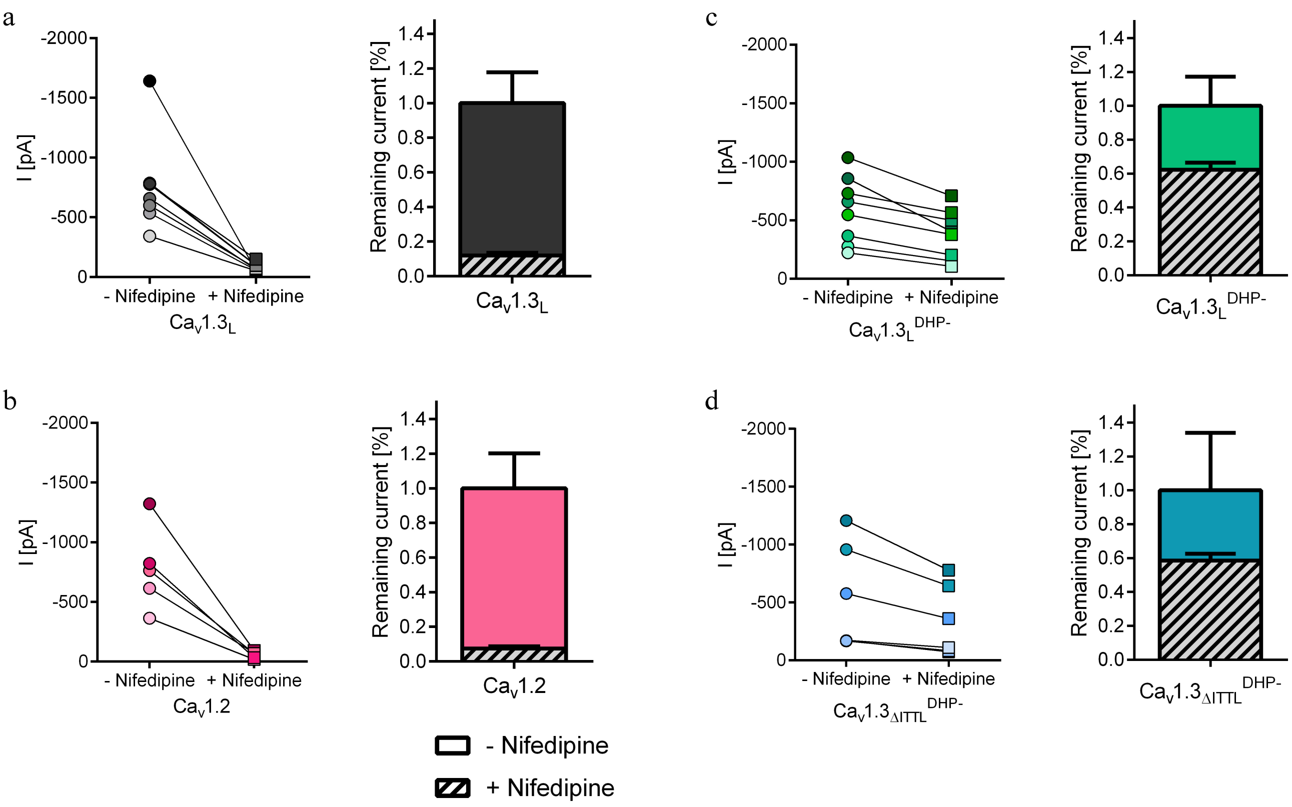

For testing the DHP sensitivity wildtype $Ca_V1.3_L$ (***a***), $Ca_V1.2$ (***b***), and the DHP-insensitive mutants $Ca_V1.3_L^{DHP-}$ (***c***) and $Ca_V1.3_{\Delta ITTL}^{DHP-}$ (***d***) channel constructs plus β_{4b}, $\alpha_2\delta$-1, and eGFP were expressed in tsA-201 cells and a bath solution containing 30 µM nifedipine was applied after at least four constant control sweeps during perfusion with bath solution only. To assess the effect of 30 µM nifedipine on different constructs, cells were depolarized from a holding potential of -50 mV to V_{max} for 100 ms at 0.1 Hz and peak currents before and 30 s after nifedipine application, which was sufficient to reach steady-state inhibition, were analyzed. Left panels: Peak currents for each individual cell (dots connected by a solid line) before and after nifedipine application; right panels: Fraction of remaining currents after nifedipine application normalized to the peak currents before nifedipine application [mean±sem]. Peak currents were reduced to 12% ($Ca_V1.3_L$, n=8), 62% ($Ca_V1.3_L^{DHP-}$, n=8), 59% ($Ca_V1.3_{\Delta ITTL}^{DHP-}$, n=6), and 7% ($Ca_V1.2$, n=5). Differences in remaining current were highly significant between $Ca_V1.3_L$ and the DHP-insensitive mutants $Ca_V1.3_L^{DHP-}$ and $Ca_V1.3_{\Delta ITTL}^{DHP-}$ ($p<0.001$), but not between $Ca_V1.3_L^{DHP-}$ and $Ca_V1.3_{\Delta ITTL}^{DHP-}$ ($p<0.85$). ANOVA with Holm-Sidak posthoc analysis, $F_3=81.8$, p<0,001.
